# Reirradiation of recurrent glioblastoma: Results from a single-center retrospective cohort study^☆^

**DOI:** 10.1016/j.ctro.2025.101029

**Published:** 2025-08-08

**Authors:** Cas S. Dejonckheere, Thomas Zeyen, Cathrina Duffy, Yannik C. Layer, Anna-Laura Potthoff, Barbara D. Wichtmann, Lea L. Friker, Davide Scafa, Christina Leitzen, Younèss Nour, Fabian Kugel, Niklas Schäfer, Alexander Radbruch, Hartmut Vatter, Anca-Ligia Grosu, Ulrich Herrlinger, Matthias Schneider, Frank A. Giordano, Gustavo R. Sarria, Eleni Gkika, Julian P. Layer

**Affiliations:** a Department of Radiation Oncology, University Hospital Bonn, Bonn, Germany; b Department of Neurooncology, Center for Neurology and Integrated Oncology (CIO), University Hospital Bonn, Bonn, Germany; c Department of Radiology, University Hospital Bonn, Bonn, Germany; d Department of Neurosurgery, University Hospital Bonn, Bonn, Germany; e Department of Neuroradiology, University Hospital Bonn, Bonn, Germany; f Institute of Neuropathology, University Hospital Bonn, Bonn, Germany; g Institute of Experimental Oncology, University Hospital Bonn, Bonn, Germany; h Department of Radiation Oncology, University Medical Center Freiburg, Faculty of Medicine, University of Freiburg, Freiburg, Germany; i Department of Radiation Oncology, University Medical Center Mannheim, Mannheim, Germany; j DKFZ-Hector Cancer Institute, University Medical Center Mannheim, Mannheim, Germany; k Mannheim Institute of Intelligent Systems in Medicine (MIISM), Medical Faculty Mannheim, Heidelberg University, Mannheim, Germany

**Keywords:** Glioblastoma, Recurrence, Reirradiation, Radiation necrosis

## Abstract

**Purpose:**

The management of recurrent glioblastoma (rGBM) remains a clinical challenge, with only limited therapeutic options available to date. Reirradiation may offer a progression-free survival (PFS) benefit in selected cases, but data are scarce.

**Methods:**

Consecutive patients from the last 10 years with GBM (CNS WHO grade 4, IDH-wildtype) who underwent at least one additional course of cranial radiotherapy for suspected or histopathologically confirmed rGBM at a tertiary neuro-oncological center were retrospectively analyzed. The primary endpoint was PFS, secondary endpoints included reirradiation-related adverse event rates, with a particular focus on radiation necrosis (RN).

**Results:**

Fifty-nine patients were included with a median follow-up (range) of 8.7 (0.5–48.0) months after reirradiation. The median time to first recurrence was 15 (4–89) months, with the majority occurring in-field (59.7 %). The EQD2_⍺/β=10_ ranged from 31.3–80.2 Gy with a median prescription dose of 42 Gy. Reirradiation was combined with systemic therapy in 81.4 % of patients. No grade 3–5 acute reirradiation-related adverse events were observed. RN was diagnosed in 16.9 % of patients (80 % grade 2 and 20 % grade 3), with a notably low rate in those receiving anti-VEGF therapy parallel to reirradiation. RN risk was independent of reirradiation volume or dose (*p* = 0.15 and 0.43, respectively). The disease control rate following reirradiation was 83.6 % and the median PFS was 5.9 (0.5–48.0) months. Concomitant chemotherapy or anti-VEGF therapy was significantly associated with improved outcomes (*p* = 0.049), whereas smaller reirradiation volumes demonstrated a non-significant trend towards longer PFS (*p* = 0.23).

**Conclusion:**

In this retrospective analysis, reirradiation for rGBM was feasible and safe, conferring a potential PFS benefit in selected patients. Bevacizumab emerged as a particularly promising combination partner, contributing to both RN prevention and enhanced efficacy.

## Highlights

• Reirradiation for rGBM was safe with no grade 3–5 acute adverse events.
• Radiation necrosis (RN) occurred in 16.9% of patients, mostly grade 2.
• Anti-VEGF therapy reduced RN risk and improved treatment efficacy.
• Concomitant systemic therapy was linked to significantly longer PFS.
• No benefit for dose intensification beyond current recommendations.

## Introduction

Glioblastoma (GBM) is the most aggressive primary brain tumor in adults, characterized by rapid progression and a dismal prognosis. [1] Despite multimodal treatment approaches—including surgical resection, radiotherapy, and chemotherapy—recurrence is almost inevitable. [2] The management of recurrent GBM (rGBM) remains a clinical challenge, with only limited therapeutic options available to date. [3].

Reirradiation involves delivering an additional course of radiotherapy within or adjacent to a previously irradiated field. [4] Initial reports in the context of glioma date back several decades. [5] Recent technical advancements, however, have led to a renewed interest. A systematic review conducted by the European Society of Radiotherapy and Oncology (ESTRO) and the European Organization for Research and Treatment of Cancer (EORTC) found that one-quarter (117/439) of reirradiation studies published between 2000 and 2020 involved the brain (mostly in the context of high-grade glioma). [4] Retrospective studies, using very heterogeneous dose-fractionation schedules, have demonstrated a range of outcomes, with overall survival (OS) up to one year following reirradiation. [6] Factors such as performance status, time to initial progression, biologically effective dose (BED; calculated on the prescribed dose) of the reirradiation, concomitant systemic therapy, and tumor volume at recurrence have been identified as potential prognostic factors. [[7], [8], [9]] There exists, however, no standard of care for target volume delineation nor dose prescription for reirradiation of rGBM. NRG Oncology/RTOG1205, the first prospective randomized trial in this setting, demonstrated a clinically meaningful improvement in progression-free survival (PFS), but not OS, for the addition of reirradiation (35 Gray in 10 fractions, using modern radiotherapy techniques) to bevacizumab in rGBM. [10].

Recently, the ESTRO and the European Association of Neuro-Oncology (EANO) published a recommendation on reirradiation in the context of rGBM, in an effort to standardize practice. [11] Based on the available prospective evidence and expert opinions, aspects such as patient selection, imaging, target volume delineation, and dose-fractionation are discussed. Here, we present a retrospective analysis of a single-center cohort of patients who underwent reirradiation for rGBM. Our study aims to evaluate outcome, identify prognostic factors, and thus contribute to the growing body of evidence supporting the role of reirradiation in the management of rGBM.

## Materials and Methods

### Patient selection

**Fig. 1:**
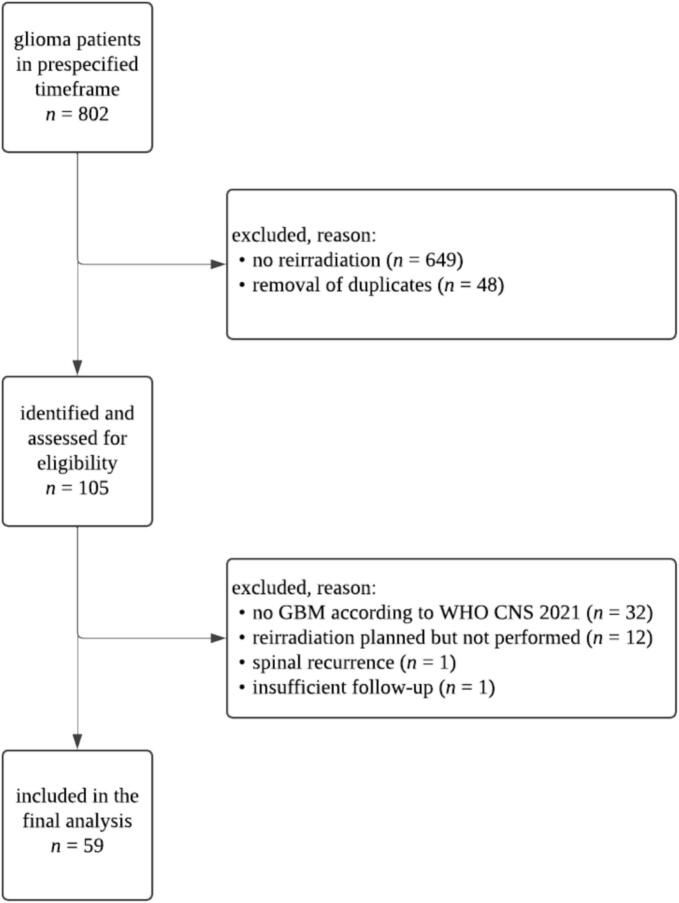
Overview of patient selection. Patients were included if they had received radiotherapy in the primary setting and at least one additional course of cranial radiotherapy for suspected or histopathologically confirmed recurrent glioblastoma (*n* = 59). GBM: glioblastoma; WHO: World Health Organization; CNS: central nervous system.

The institutional database of the Department of Radiation Oncology at our tertiary neuro-oncological center was searched for consecutive cases with GBM (according to the currently valid World Health Organization [WHO] Classification of Tumors of the Central Nervous System [CNS]) in the timeframe between January 2015 and February 2025, using structured query language (SQL). [12] Patients were included if they had received radiotherapy in the primary setting and at least one additional course of cranial radiotherapy for suspected or histopathologically confirmed rGBM. Both in-field and out-of-field recurrences were included, with their definition depending on the relation between the initial planning target volume (PTV; 100 % isodose line) and the reirradiation gross tumor volume (GTV). In the case of overlap, recurrences were classified as marginal. Data on initial and salvage therapies were extracted from individual patient records and digital radiation treatment plans (Eclipse version 17, Varian Medical Systems, Palo Alto, CA, USA). An overview of patient selection is presented in Fig. 1. This retrospective study was approved by the Institutional Review Board of the University Hospital Bonn, Germany (2025–43-BO).

### Reirradiation

Following discussion in an interdisciplinary neuro-oncological tumorboard, reirradiation was offered to patients with rGBM on an individual basis. Upon informed consent, all patients received a planning computed tomography (CT) in a supine position with an individual thermoplastic fixation mask. Target volume delineation was performed according to the prevailing standards at the time of reirradiation. A contrast-enhanced planning magnetic resonance imaging (MRI) with 1 mm-slice thickness was coregistered with this planning CT in all patients. If available, information derived from an amino acid positron emission tomography (AA-PET; with O-(2-([18]F]fluoroethyl)-L-tyrosine as a tracer in all cases) at the time of rGBM was considered as well, however, no biological tumor volume (BTV) was defined. The extent of the margin from the GTV to the clinical target volume (CTV) was dependent upon the overall volume, dose/fractionation, and pattern of recurrence. In patients with a complete out-of-field recurrence, a margin up to 15 mm was employed (as in the primary setting). All CTVs were edited to take anatomical barriers to tumour spread into account. The isotropic margin to generate the PTV ranged from 1–5 mm depending on the fixation system and planned daily image-guidance modality, as per institutional standards. Reirradiation was planned with intensity-modulated techniques, employing 6 − 10 MV photon energies and striving for a target volume coverage of 95 − 107 % or 99 − 120 %, following the respective International Commission on Radiation Units and Measurements (ICRU) recommendations. All patients were treated on a TrueBeam STx (Varian Medical Systems, Palo Alto, CA, USA) linear accelerator, using either the on-board cone beam CT system or ExacTrac (Brainlab, Munich, Germany) for position matching.

### Follow-Up and endpoints

In accordance with institutional standards, the first follow-up MRI was performed six weeks after reirradiation and every three months subsequently. Follow-up visits also included a neurological examination. Radiotherapy-related adverse events were assessed and graded by clinicians according to the National Cancer Institute’s (NCI) Common Terminology Criteria for Adverse Events (CTCAE; version 5.0). [13] Acute toxicities were defined as occurring within the first three months after completion of reirradiation, whereas late complications were defined as all adverse events recorded at a later timepoint. MRI reporting was performed by board-certified neuroradiologists, according to the modified Response Assessment in Neuro-Oncology (mRANO) criteria. [14] The PFS event was defined as the first date on which predefined progression criteria had been met, i.e. the date of the sequentially confirmed MRI assessment showing preliminary progressive disease (PD) according to mRANO, the date of initiation of a further-line therapy, or the date of death by any cause if the patient died before clinical or radiological progression. PFS was defined as the interval between the first day of reirradiation and the PFS event. OS was calculated as the time from initial surgery until death by any cause and post-reirradiation survival (PRS) from the first day of reirradiation until death by any cause. If lost to follow-up, patients were censored at the time of the last documented patient contact. Upon uncertainty regarding clinical or radiological progression, the interdisciplinary neuro-oncological tumorboard was consulted and, if indicated, additional advanced imaging techniques such as perfusion sequences, dynamic susceptibility contrast (DSC) MRI, or AA-PET were indicated. In some cases, additional histological confirmation was sought. Radiation necrosis (RN) in particular was investigated as a potential late complication of reirradiation. RN was defined as any of the following conditions: (1) after initial suspected PD, at least two follow-up MRI timepoints showed no sign of PD, (2) advanced MRI incorporating DSC and diffusion-weighted imaging (DWI) were suggestive of RN, or (3) RN was confirmed histopathologically. RN grading also followed CTCAE version 5.0. [13].

### Statistical analysis

The primary endpoint was PFS in patients who underwent reirradiation for rGBM. Secondary endpoints included reirradiation-related adverse event rates, of RN in particular, as well as OS and PRS. Patients that were lost to follow-up or deceased prior to radiographic progression were censored at the last follow-up timepoint. The Mann-Whitney test was used to determine significance, if not stated otherwise, with the threshold set at *p* < 0.05. The χ^2^ test was performed to assess the significance of contingency tables. For the statistical comparison of high and low variable values, the cohort was divided into the respective groups by its median. For the statistical assessment of survival rates, the log-rank test was used and data presented according to the Kaplan-Meier method. Data analysis was performed with GraphPad Prism version 10 (GraphPad Software, San Diego, CA, USA). Figures and graphs were created using GraphPad Prism and Adobe Illustrator 2023 (Adobe Inc., Mountain View, CA, USA).

## Results

### Primary treatment

Fifty-nine patients met the inclusion criteria and were analyzed (Fig. 1). Of these, 44.1 % were female. The median age (range) at the time of initial GBM diagnosis was 59 (33–80) years and the median Karnofsky performance score (KPS) 90 (70–100). 10.2 % of patients had multifocal disease upon diagnosis and the temporal lobe was the most common location (40.7 %), followed by the frontal (28.8 %) and parietal (16.9 %) lobes. Gross total resection (GTR; i.e. complete resection of contrast-enhancing tumor) was achieved in 76.3 % of patients, whereas 11.9 % each had subtotal resection (STR; i.e. residual contrast-enhancing tumor) or biopsy only. All patients had histologically confirmed GBM according to the currently valid CNS WHO criteria, i.e. CNS WHO grade 4, isocitrate dehydrogenase (IDH) wildtype. Four patients (6.8 %) had primary gliosarcoma subtype and one patient (1.7 %) had giant cell GBM subtype. [15] 42.4 % of patients were O-6-methylguanine-DNA methyltransferase (*MGMT*) promoter hypermethylated. 94.4 % of patients underwent standard normofractionated radiotherapy, while 5.6 % received hypofractionated treatment with 15 fractions. Radiotherapy was combined with any concomitant systemic therapy in 96.6 % of patients. Temozolomide (TMZ) was the most common first-line systemic therapy, applied in 81.4 % of patients and combined with lomustine in 27.1 %. The median number of adjuvant systemic therapy cycles was 6 (0–6) cycles. Patient and treatment characteristics are summarized in Table 1.

**Table 1:** Summary of patient and treatment characteristics at the time of initial diagnosis (*n* = 59). KPS: Karnofsky performance score; GTR: gross total resection; STR: subtotal resection; IDH: isocitrate dehydrogenase; CNS: central nervous system; WHO: World Health Organization; *MGMT*: O-6-methylguanine-DNA methyltransferase; Gy: Gray.

**Characteristic**	***n* (%)**
Sex	
Female	33 (44.1)
Male	26 (55.9)
Median age (range) in years	59 (33–80)
Median KPS (range)	90 (70–100)
Multifocal disease	6 (10.2)

Location	
Temporal	24 (40.7)
Frontal	17 (28.8)
Parietal	10 (16.9)
Central	4 (6.8)
Occipital	3 (5.1)
Cerebellar	1 (1.7)

Surgical resection	
GTR	45 (76.3)
STR	7 (11.9)
Biopsy	7 (11.9)
Glioblastoma, IDH-wildtype (CNS WHO grade 4)	59 (100)
Gliosarcoma	4 (6.8)
Giant cell glioblastoma	1 (1.7)
*MGMT* promoter hypermethylated	25 (42.4)

Radiotherapy	
Normofractionated (59.4–60 Gy)	56 (94.4)
Hypofractionated (40–45 Gy)	3 (5.1)
Any concomitant systemic therapy	57 (96.6)
Temozolomide monotherapy	32 (54.2)
Temozolomide + lomustine	16 (27.1)
Median number of adjuvant cycles (range)	6 (0–6)

### Recurrence treatment

The median time to first recurrence was 15 (4–89) months. The majority of patients (81.4 %) had a unifocal recurrence. Of all recurrences, 59.7 % were in-field, 20.8 % were out-of-field, and 19.4 % marginal. To identify recurrence and delineate the GTV, an additional AA-PET was performed in 8.5 % of patients. The median KPS at the time of reirradiation was 80 (50–100). 18.6 % of patients underwent resurgery between both courses of radiotherapy. While the majority of patients received reirradiation at first recurrence, five patients (8.5 %) had reirradiation at the time point of second recurrence. In total, 70 lesions underwent reirradiation, with a median PTV of 18.2 (0.7–284.1) cm^3^, translating into a median proportion of total brain tissue of 1.2 % (0.1–19.7 %). A CTV margin was applied in 23.7 % of patients, with a median of 5 (3–15) mm. 35 Gy in 10 fractions (EQD2_⍺/β=2_ 48.1 Gy, BED 96.3 Gy; EQD2_⍺/β=10_ 39.4 Gy, BED 47.3 Gy) was most commonly prescribed (15.7 %), followed by 30 Gy in 6 fractions (EQD2_⍺/β=2_ 52.5 Gy, BED 105 Gy; EQD2_⍺/β=10_ 37.5 Gy, BED 45.0 Gy) and 39 Gy in 13 fractions (EQD2_⍺/β=2_ 48.8 Gy, BED 97.5 Gy; EQD2_⍺/β=10_ 42.3 Gy, BED 50.7 Gy) (14.3 % each). The EQD2_⍺/β=10_ ranged from 31.3–80.2 Gy with a median prescription dose of 42 Gy. Single-fraction radiosurgery was used for 15.7 % of recurrent lesions, with doses ranging from 15–20 Gy. Reirradiation was combined with systemic therapy in 81.4 % of patients, most commonly TMZ (32.2 %), lomustine (22.0 %), bevacizumab (15.3 %), and regorafenib (5.1 %). Six patients (10.2 %) underwent a second course of reirradiation during follow-up. Patient and treatment characteristics at the time of reirradiation are summarized in Table 2, dosimetric properties of reirradiation are summarized in Table 3.

**Table 2:** Summary of patient and treatment characteristics at the time of reirradiation (*n* = 59 patients with *n* = 70 irradiated lesions). PTV: planning target volume; PET: positron emission tomography; KPS: Karnofsky performance score; CTV: clinical target volume; Gy: Gray.

**Characteristic**	***n* (%)**
Median time to first recurrence (range) in months	15 (4–89)
Solitary recurrence	48 (81.4)
Total number of reirradiated lesions	70
Median number of reirradiated lesions (range)	1 (1–3)

Location with respect to initial PTV	
In-field	43 (59.7)
Out-of-field	15 (20.8)
Marginal	14 (19.4)
Amino acid PET performed	5 (8.5)
Median KPS (range)	80 (50–100)
Resurgery prior to reirradiation	11 (18.6)

Reirradiation timepoint	
1st recurrence	54 (91.5)
2nd recurrence	5 (8.5)
Median PTV (range) in cm^3^	18.2 (0.7–284.1)
Median proportion of total brain tissue (range) in cm^3^	1.2 (0.1–19.7)
Use of CTV margin	14 (23.7)
Median CTV margin (range)	5 (3–15)

Reirradiation dose prescription (EQD2_⍺/β=10_)	
10 × 3.5 Gy (39.4 Gy)	11 (15.7)
1 × 15–20 Gy (31.3–50.0 Gy)	11 (15.7)
6 × 5 Gy (37.5 Gy)	10 (14.3)
13 × 3 Gy (42.3 Gy)	10 (14.3)
15 × 3 Gy (46.9 Gy)	6 (8.6)
7 × 5 Gy (43.8 Gy)	5 (7.1)
15 × 2.67 Gy (42.2 Gy)	4 (5.7)
5 × 5 Gy (31.3 Gy)	3 (4.2)
Other	10 (14.3)
Any concomitant systemic therapy	48 (81.4)
Temozolomide	19 (32.2)
Lomustine	13 (22.0)
Bevacizumab	9 (15.3)
Regorafenib	3 (5.1)
Other	4 (6.8)

**Table 3:** Dosimetric properties of reirradiation.

**Characteristic**	**Median (range) in Gy**
Cumulative Dmax	94.5 (59.5–111.6)
Cumulative Dmean brain	28 (13.6–46.0)
Cumulative Dmax brainstem	59.5 (4.0–95.9)
Cumulative Dmax optic chiasm	40.4 (3.8–69.1)

### Toxicity

Regarding radiation-related toxicity, 36 acute adverse events were documented (*Supplementary* Table 1): 77.8 % were grade 1 and 22.2 % grade 2. No higher-grade acute events were observed. Fifteen late effects were reported (*Supplementary* Table 1): 26.7 % grade 1, 53.3 % grade 2, and 20.0 % grade 3. RN occurred in 9 patients (15.3 %; 7 grade 2 events and 2 grade 3 events), accounting for 60.0 % of all late events. RN-free survival at 6- and 12-months was 88.2 and 80.3 %, respectively (*Supplementary Fig. 1*). Using the median prescription dose (EQD2_⍺/β=10_ 42 Gy) as a classifier, no significant correlation between RN and prescription dose was observed (*p* = 0.43). Greater PTV extent showed a trend towards, but was not significantly associated with RN events (*p* = 0.15). Of note, although not statistically significant compared to other systemic therapies due to the low number of events (*p* = 0.16), RN occurred in only one of the three patients receiving the vascular endothelial growth factor (VEGF) receptor-targeting multikinase inhibitor regorafenib concomitantly and in none of the nine treated with bevacizumab. Median time to onset of RN was 3.5 (1–13) months and diagnosis was based on MRI in all patients and additional AA-PET in 2 patients. To treat RN, all patients received bevacizumab, achieving sufficient symptom control and edema size reduction.

### Outcome

**Fig. 2:**
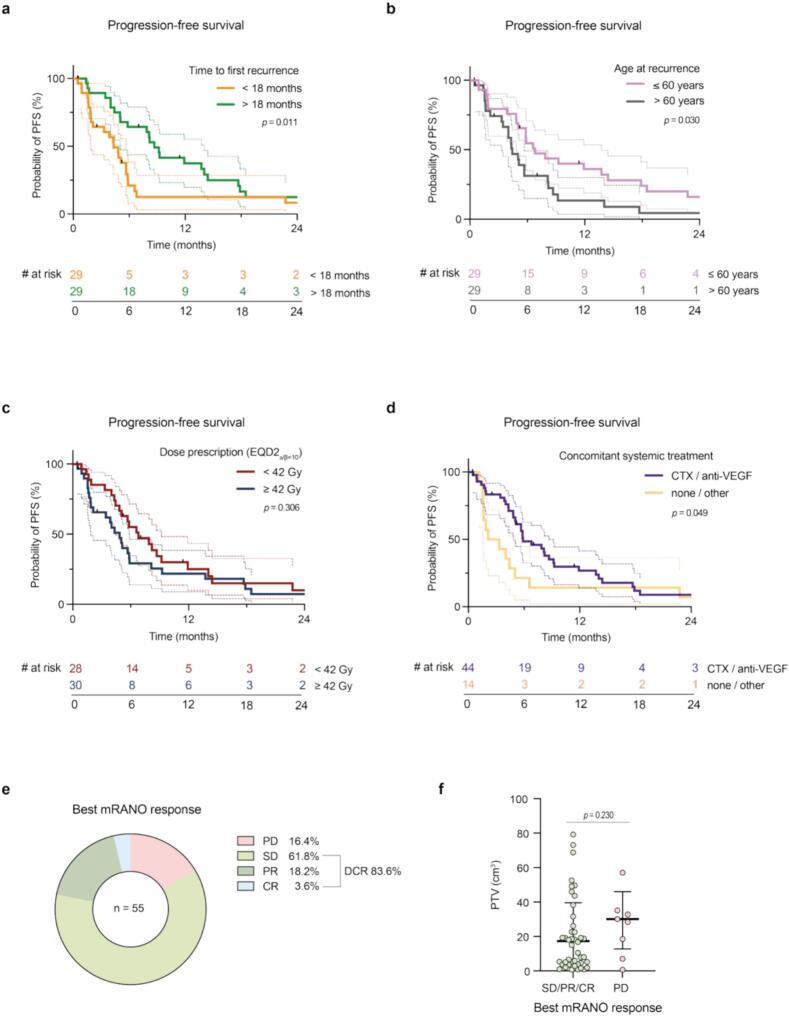
Clinical outcome after reirradiation for recurrent glioblastoma. **(a, b, c, d)** Kaplan-Meier curves of progression-free survival (PFS) in the first 24 months depending on **(a)** time to initial progression < 18 months (orange) versus > 18 months (green), **(b)** age at recurrence ≤ 60 years (pink) versus > 60 years (gray), **(c)** dose prescription in EQD2_⍺/β=10_ < 42 Gy (red) versus ≥ 42 Gy (blue) as well as **(d)** concomitant administration of systemic treatment (chemotherapy or VEGF-targeting agents in purple versus none or other treatments in yellow). Log-rank test (two-tailed); dashed lines indicate 95 % confidence intervals; *p*-values are shown in the corresponding graphs. **(e)** Distribution of best radiographic tumor response after reirradiation as per mRANO. **(f)** Dot plot showing reirradiation PTV sizes in cm^3^ (up to 100 cm^3^) depending on radiographic tumor response as per mRANO (green: SD, PR, CR; purple: PD). Mann-Whitney test; *p*-values are depicted in the graph. VEGF: vascular endothelial growth factor; mRANO: modified Response Assessment in Neuro-Oncology; PTV: planning target volume; SD: stable disease; PR: partial response; CR: complete response; PD: progressive disease; CTX: chemotherapy; DCR: disease control rate. (For interpretation of the references to colour in this figure legend, the reader is referred to the web version of this article.)

The median follow-up after reirradiation was 8.7 (0.5–48.0) months for the whole cohort. Herein, patients received a median number of 3 (0–14) MRIs. The median PFS was 5.9 (0.5–48.0) months. PFS after reirradiation was significantly associated with the time to initial progression (*p* = 0.01; Fig. 2*a*; cut-off 18 months) and age at recurrence (*p* = 0.03; Fig. 2*b*; cut-off 60 years*),* but not KPS at recurrence (*p* = 0.75). With a median PFS of 5.0 versus 8.7 months, there was no significant trend towards improved PFS for patients undergoing resurgery prior to reirradiation (*p* = 0.41). Equally, there was no significant difference in PFS of patients with a reirradiation prescription dose < 42 Gy (EQD2_⍺/β=10_) and those receiving ≥ 42 Gy (*p* = 0.31; Fig. 2*c*). While median PFS was 7.9 months and 5.7 months for patients receiving concomitant chemotherapy and anti-VEGF-therapy, respectively, patients receiving non-standard agents or no concomitant systemic treatment had a significantly inferior median PFS of 2.8 and 2.7 months, respectively (*p* = 0.049; Fig. 2*d*). The corresponding PRS did, however, not differ significantly (*p* = 0.39). Of all patients with at least one follow-up MRI (*n* = 55; 93.2 %), the best mRANO response was PD in 16.4 %, stable disease in 61.8 %, partial response in 18.2 % and complete response in 3.6 % of cases, thus resulting in a disease control rate of 83.6 % (Fig. 2*e*). There was a non-significant trend towards better response with smaller PTV size at the time of reirradiation (*p* = 0.23; Fig. 2*f*). The median OS was 39.0 (7.5–119.7) months and the median PRS 10.7 (0.5–48.0) months (*Supplementary Fig. 2*). Of the variables previously assessed for PFS, only the time to initial progression was significantly associated with an improvement (*p* = 0.006). A tabular overview of the survival analyses (PFS and PRS) is provided in *Supplementary* Table 2.

## Discussion

Reirradiation has emerged as a viable treatment option in selected patients with rGBM, aiming to delay tumor progression and potentially extend survival in the context of an otherwise dismal prognosis. Despite growing clinical adoption, high-level evidence remains scarce, with the ESTRO/EANO recommendations relying primarily on a few moderate-quality studies, largely due to the lack of prospective randomized trials. [11] To bridge this gap, additional retrospective analyses are needed to solidify the existing evidence and provide a basis for future prospective trials. In this retrospective single-center analysis, we evaluated imaging strategies, clinical outcomes, and prognostic factors associated with reirradiation in a homogeneously selected cohort, aiming to contribute to the evolving framework for rGBM management.

We most commonly observed initial recurrences in-field, in line with previous reports. [16] This supports the trend towards smaller margins in the primary setting, aiming to reduce the exposure of normal brain tissue, improving salvage reirradiation options in case of rGBM and potentially reducing the RN risk. Target volume delineation was guided by advanced MRI techniques in all patients, reflecting institutional practices. However, AA-PET was performed in 9 % of patients only. Its higher specificity and potential to differentiate between treatment-related changes (RN in particular) and true recurrence have led to the rationale of implementing AA-PET into radiotherapeutic (re-)treatment planning. [17] In the primary setting, AA-PET has been shown to identify a higher number of patients with measurable disease in comparison with standard MRI. [18] Small series suggest that AA-PET reveals a more complete extent of tumor infiltration in recurrent glioma, thus questioning the practice of using an isotropic CTV margin around the MRI-based GTV, as recurrence after reirradiation was significantly higher within AA-PET volume in comparison with the MRI-based one. [19] The GLIAA trial, however, randomizing between MRI- or AA-PET-based target volume delineation for reirradiation in rGBM (13 × 3 Gy), failed to demonstrate an oncological benefit of the AA-PET-based approach. [20] While the question of optimal target volume delineation for rGBM remains unanswered at this time, our data confirm the safety of a small CTV margin on an individual basis, particularly considering the overall PTV size and planned dose prescription, as a trend towards a higher RN risk was observed.

In our cohort, a recommended reirradiation BED > 36 Gy in 2 Gy-fractions was achieved in 95 % of patients, with a prescribed dose of 10 × 3.5 Gy (as per NRG Oncology/RTOG1205 and BRIOChe) most commonly used. This fractionation regimen derived from the only prospective trials in the field was primarily applied in cases treated more recently, reflecting the adoption of emerging prospective evidence into daily clinical practice. While this concept proved safe and efficient and became our institutional standard for in-field-reirradiation, we did not observe a clinical benefit from differing schemes achieving higher BEDs.

Patients with rGBM may benefit from multimodality salvage treatment. In our cohort, only 19 % of patients underwent resurgery prior to reirradiation, rendering subgroup analyses stratified by resurgery status difficult. According to a report of the RANO *resect* group, resurgery, if feasible, positively impacts survival, even after stratification for clinical and molecular confounders. [21] In their multicenter cohort of 310 patients undergoing resurgery for first GBM recurrence, Karschnia *et al.* also found that non-surgical adjuvant treatment substantially extended survival after resurgery. [21] A clear recommendation regarding concomitant systemic treatment cannot be given at this time. [11] Unlike alkylating chemotherapy, reirradiation can be considered independent of age or *MGMT* methylation status. [11] In a systematic review and *meta*-analysis of 31 studies (2,084 patients; 76 % CNS WHO grade 4) by Marwah *et al.*, combination therapy improved PFS and OS compared to either reirradiation or systemic therapy alone. [22] Potential synergies between reirradiation and immune checkpoint inhibitors have been described, e.g. reirradiation combined with ipilimumab, nivolumab, and bevacizumab in a recent phase 1 trial. [23] Overcoming a cold (i.e. immunosuppressive) tumor microenvironment through immune checkpoint inhibitors has also been tested in the neoadjuvant setting as monotherapy (pembrolizumab) before resurgery or as a triplet (ipilimumab, nivolumab, relatlimab) before initial surgery. [24,25] Reirradiation plus pembrolizumab in particular seemed to improve survival among bevacizumab-refractory patients, but randomized trials are warranted. [26] In addition to immunological factors, rGBM is thought to be linked to a complex VEGF- and CXCL12-driven revascularization of the tumor microenvironment. [27] Therefore, antiangiogenic substances have been tested widely in the context of GBM, both in the first-line setting and at recurrence. [[28], [29], [30]] Our work underscores the potential of bevacizumab as a combination partner for reirradiation of rGBM, both in RN prevention and treatment, while potentially providing a PFS benefit for patients, in line with previous reports. [[31], [32], [33], [34], [35]] Patients appear to benefit from concomitant systemic treatment in terms of tumor control, while not suffering from a greater risk of reirradiation-related side effects. Systemic toxicity, however, remains a factor to be considered in careful patient selection.

Regarding patient selection, 93 % had a KPS ≥ 60 and all patients had an interval of at least 6 months between both courses of radiation, in alignment with the recent ESTRO/EANO recommendations. [11] Prognostic factors for rGBM reported by other studies include age, KPS, time to initial progression, reirradiation PTV size, reirradiation BED, *MGMT* promoter status, and concomitant systemic therapy (Table 4), some of which are corroborated by our data. However, the potential selection bias associated with these factors is evident when comparing reirradiation cohorts with these of general GBM cases, suggesting that reirradiation may not be a feasible or effective option for all patients. We refrained from performing subgroup analysis by *MGMT* promoter status, as reirradiation may be considered independent of this result by current recommendations.

**Table 4:** Overview of selected similar contemporary studies on reirradiation for recurrent glioma. CNS: central nervous system; WHO: World Health Organization; mFU: median follow-up; PFS: progression-free survival; PRS: post-reirradiation survival; RN: radiation necrosis; KPS: Karnofsky performance score; nr: not reported; BED: biologically effective dose; PTV: planning target volume; *MGMT*: O-6-methylguanine-DNA methyltransferase; GTR: gross total resection.

**Author (year)**	***n***	**CNS WHO grade 4 (%)**	**mFU**	**PFS**	**PRS** *	**Prognostic factors**	**RN (%)**
**Calduch (2024)** [38]	44	100	15	5	15	OS: KPS, size of recurrence	22
**Chapman (2019)** [7]	116	85	nr	5	11	PFS: KPS, time to initial progression, BED OS: age, time to initial progression, PTV	7 (at 1 year) ***
**Ehret (2023)** [39]	88	100	5	6	8	OS: GTR before reirradiation	0
**González (2024)** [40]	52	42	nr	5	12	PFS: 5 × 6 Gy	4 ***
**Kaul (2020)** [41]	198	67	7	nr	7	OS: age, KPS	nr
**Kite (2025)** [42]	33	82	6	6	7	OS: *MGMT*	3
**Rogers (2024)** [9]	50	100	9	7	12	PFS: concomitant systemic therapy	9
**Shen (2018)** [43]	118	74	7	nr	10	OS: time to initial progression, BED, GTR before reirradiation	5
**Straube (2019)** [44] **	25	100	nr	4	7	OS: time to initial progression	0
**Yilmaz (2024)** [8]	77	100	9	8	10	PFS: BED, presence of pseudoprogression, time to initial progression OS: BED, presence of pseudoprogression	22
**Current (2025)**	59	100	9	6	11	PFS: time to initial progression, age, concomitant systemic therapy PRS: time to initial progression	15

* Entire cohort of patients, regardless of CNS WHO grade.

** Only elderly patients (i.e. > 65 years old) were included.

*** Reported symptomatic RN only.

Our study is not without limitations, primarily its retrospective nature, relatively small sample size, and heterogeneity in dose prescriptions. The absence of uniform treatment regimens in our cohort reflects the ongoing lack of consensus and concise evidence-based practice guidelines for reirradiation in rGBM. Besides the factors discussed above, a relatively high proportion of patients achieved GTR and underwent normofractionated adjuvant radiotherapy in the primary setting, which might have introduced selection bias. However, we reason that this reflects the real-world population considered for reirradiation at a high-volume tertiary neuro-oncological referral center. It appears plausible that only this positively selected patient subgroup with inherently longer survival remains eligible for reirradiation. The homogeneous collective in terms of histopathological diagnosis according to the CNS WHO classification adds to the existing body of evidence of reirradiation for rGBM, this distinguishes our study from other cohorts that often included mixed histologies (Table 4). The lack of histopathological confirmation of rGBM in many cases—either due to inoperability or patient status—remains a major limitation in retrospective series, introducing uncertainty about the true nature of the recurrence (tumor progression versus treatment effect). The use of advanced MRI sequences with closely repeated follow-ups along with AA-PET for specific indications at our center may aid in mitigating this limitation.

Ongoing trials will further elucidate the preferred approach for rGBM, investigating multimodality treatments in the majority of cases. The LEGATO trial (EORTC-2227-BTG; NCT05904119) investigates lomustine with or without reirradiation for first progression of GBM. [36] The GlioCave trial (NOA-17; NCT02715297) randomizes between observation or reirradiation (23 × 2 Gy or 12 × 3 Gy depending on tumor size) after GTR for rGBM. [37] The development of prospective, histologically homogeneous, and biomarker-integrated trials remains an urgent priority within the reirradiation community.

## Conclusion

Our data support the emerging evidence on the feasibility and safety of reirradiation in carefully selected patients with rGBM. While reirradiation remains an important tool in the salvage setting, multidisciplinary integration with surgery, advanced imaging techniques, and systemic therapies—including antiangiogenic agents—may further improve outcomes. However, prospective trials are essential to guide evidence-based decision-making in this challenging clinical scenario.

## Funding

The authors declare that no funds, grants, or other support were received during the preparation of this manuscript.

## Declaration of Competing Interest

The authors declare the following financial interests/personal relationships which may be considered as potential competing interests: FAG reports travel expenses, stocks, and honoraria from TME Pharma AG; research grants and travel expenses from ELEKTA AB; grants, research grants, travel expenses, and honoraria from Carl Zeiss Meditec AG; grants, research grants, travel expenses, and honoraria from OncoMANGETx, Inc.; travel expenses and research grants from Varian Medical Systems, Inc.; travel expenses and/or honoraria from Bristol-Myers Squibb, Cureteq AG, Roche Pharma AG, MSD Sharp and Dohme GmbH, Siemens Healthineers AG, Varian Medical Systems, Inc., and AstraZeneca GmbH; non-financial support from Oncare GmbH and Opasca GmbH; patent US10857388B2 together with Carl Zeiss Meditec AG, and patent EP4119191A1 with the University of Heidelberg. The remaining authors report no conflict of interest related to this work
