# Supplementary material for: Reirradiation of recurrent glioblastoma: Results from a single-center retrospective cohort study
Source: Clin Transl Radiat Oncol. 2025 Aug 8;55:101029. doi: 10.1016/j.ctro.2025.101029 (PMC12357250; doi:10.1016/j.ctro.2025.101029)
Supplement: Supplementary Data 1 [file mmc1.docx]

**Supplement**

**Supplementary Figure 1.** Radiation necrosis-free survival (in months) after reirradiation for recurrent glioblastoma.


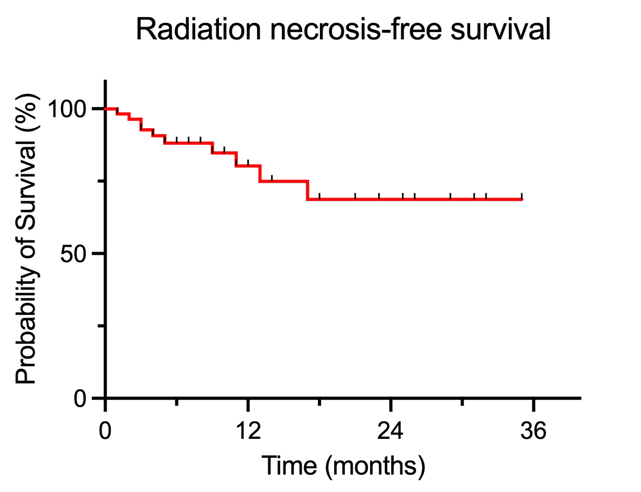


**Supplementary Figure 2.** **(a)** Post-reirradiation survival and **(b)** overall survival of the study cohort. Kaplan-Meier curves; dashed lines indicate 95% confidence intervals.


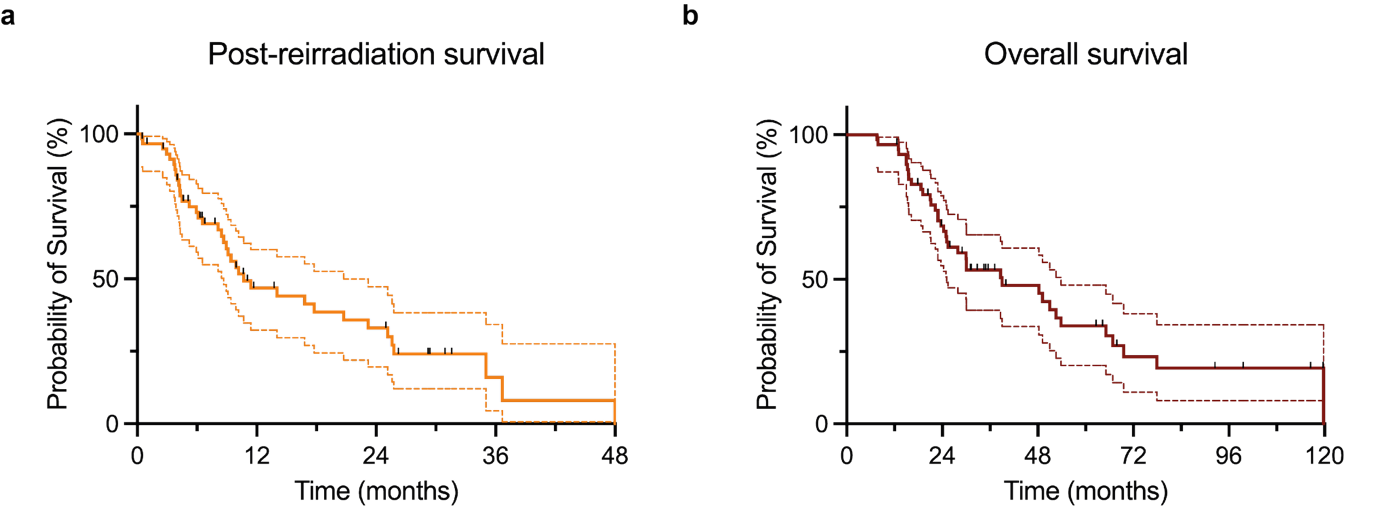


**Supplementary Table 1.** Acute and late reirradiation-related adverse events according to the Common Terminology Criteria for Adverse Events (CTCAE; version 5.0).

| **Acute adverse events** | ***n* (%)** |
| --- | --- |
| Any  Any grade  Grade 1  Grade 2  Grade 3–5 | 36 (100)  28 (77.8)  8 (22.2)  0 (0) |
| Fatigue  Any grade  Grade 1  Grade 2 | 15  12  3 |
| Headache  Any grade  Grade 1  Grade 2 | 12  9  3 |
| Alopecia (grade 1) | 4 |
| Muskle weakness (grade 2) | 1 |
| Seizure (grade 2) | 1 |
| Dizziness (grade 1) | 1 |
| Nausea (grade 1) | 1 |
| Radiation dermatitis (grade 1) | 1 |
| **Late adverse events** | ***n* (%)** |
| Any  Any grade  Grade 1  Grade 2  Grade 3  Grade 4–5 | 15 (100)  4 (26.7)  8 (53.3)  3 (20.0)  0 (0) |
| Radiation necrosis  Any grade  Grade 1  Grade 2  Grade 3 | 9  0  7  2 |
| Headache  Any grade  Grade 1  Grade 2 | 2  1  1 |
| Stroke (grade 3) | 1 |
| Alopecia (grade 1) | 1 |
| Dizziness (grade 1) | 1 |
| Fatigue (grade 1) | 1 |

**Supplementary Table 2.** Results of the subgroup survival analyses (two-tailed log-rank test, *p*-values) for the indicated variables assessed for progression-free survival (PFS) and post-reirradiation survival (PRS). KPS: Karnofsky performance score.

| **Variable** | **PFS** | **PRS** |
| --- | --- | --- |
| Time to initial progression | **0.011** | **0.006** |
| Systemic therapy | **0.049** | 0.389 |
| Age at recurrence | **0.030** | 0.112 |
| Reirradiation dose (< vs. ≥ 42 Gy EQD2_⍺/β=10_) | 0.306 | 0.208 |
| Resurgery prior to reirradiation | 0.413 | 0.253 |
| KPS at recurrence | 0.755 | 0.981 |
